# Genetic polymorphisms of genes involved in oxidative stress and inflammatory management in oncopediatric patients with chemo-induced oral mucositis

**DOI:** 10.1590/1678-7757-2021-0490

**Published:** 2022-03-23

**Authors:** Marina Castro Coêlho, José Maria Chagas Viana, Beatriz Fernandes de Souza, Ana Maria Gondim Valença, Darlene Camati Persuhn, Naila Francis Paulo de Oliveira

**Affiliations:** 1 Universidade Federal da Paraíba Centro de Ciências da Saúde João Pessoa Paraíba Brasil Universidade Federal da Paraíba - UFPB, Centro de Ciências da Saúde, Programa de Pós-Graduação em Odontologia, João Pessoa, Paraíba, Brasil.; 2 Universidade Federal da Paraíba Centro de Ciências Exatas e da Natureza Departamento de Biologia Molecular João Pessoa Paraíba Brasil Universidade Federal da Paraíba - UFPB, Centro de Ciências Exatas e da Natureza, Departamento de Biologia Molecular, João Pessoa, Paraíba, Brasil.

**Keywords:** Oral mucositis, Genetic polymorphism, Oxidative stress, Cytokines

## Abstract

Oral mucositis (OM) is a painful inflammatory oral condition that affects children who undergo chemotherapy. Oxidative stress is a known OM mediator and pro-inflammatory cytokines contribute to the amplification of the immune response.

**Objective:**

To investigate the possible associations of rs4880 (superoxide dismutase 2, *SOD2* 47 C/T), rs7943316 (catalase, *CAT* −21 A/T), rs1800629 (tumor necrosis factor α, *TNF-* α −308 G/A), and rs1800795 (interleukin 6, *IL-6* −174 G/C) polymorphisms with chemo-induced OM occurrence and severity in oncopediatric patients.

**Methodology:**

We conducted a single-center, observational cross-sectional study with sample collection of oral epithelial cells from 95 children and adolescents with hematological cancers who underwent chemotherapy, followed by genomic DNA extraction. Single-nucleotide polymorphisms (SNPs) were assessed with PCR-RFLP (Polymerase Chain Reaction-Restriction Fragment Length Polymorphism). Demographic data and information concerning OM occurrence were obtained from dental charts of the multidisciplinary oral care team. Information on OM severity was obtained from appropriately-filled Oral Assessment Guide records. Descriptive and inferential statistics were conducted with Student's T test, chi-squared test, and Fisher's exact test, with p≤0.05.

**Results:**

The mean age was 10 years-old and most patients were male individuals (57.89%). Female sex was considered a protective factor for OM occurrence (OR=4.83; CI=[1.14; 16.57]). The AA genotype for *CAT* was the most frequent amongst individuals with severe OM (p=0.04). The GA genotype for *TNF-* α was the most frequent amongst individuals without severe OM (p=0.03). For *SOD2* and *IL-6* , the most frequent genotypes were CT and GG respectively for all groups (p>0.05).

**Conclusion:**

The AA genotype for *CAT* −21 A/T was a tendency among the group with severe OM. Data on *TNF-* α −308 G/A were inconclusive. No associations were detected for *SOD2* 47 C/T and *IL-6* −174 G/C polymorphisms in oncopediatric patients with chemo-induced oral mucositis.

## Introduction

Oral mucositis (OM) is a common debilitating adverse effect of anticancer treatments characterized by erythema and ulceration of the oral mucosa. It directly affects the quality of life of oncologic patients since even the non-ulcerative forms of this disease may be painful, compromising oral functions such as chewing and swallowing.^1^ Complications of severe OM include the routine use of opioid medication, parenteral feeding dependence, and the development of systemic infections, possibly leading to treatment discontinuation.^2^

Oxidative stress plays an important role in the establishment of this condition since the initial stage of OM development involves reactive oxygen species (ROS) generation during the apoptotic events triggered immediately after the administration of a cytotoxic agent amongst epithelial basal cells.^3^ It has been reported that, in the early stages of OM progression, the concentration levels of malondialdehyde, a lipid peroxidation marker, are nearly two times higher in the oral mucosa of mice with chemo-induced OM when compared with samples extracted from healthy controls.^4^ ROS are responsible for indirectly activating the nuclear factor NF-kB pathway, of which the genetic targets comprise stress responders, such as antioxidant enzyme superoxide dismutase 2 (SOD2), pro-inflammatory cytokines tumor necrosis factor α (TNF-α), and interleukin 6 (IL-6), among others.^5^

Superoxide dismutase, alongside catalase enzymes, represents the first line of defense against oxidative damage in living cells^6^ and recombinant SOD2 proteins have been demonstrated to attenuate chemo-induced intestinal mucositis in mice.^7^ Regarding the cytokines TNF-α and IL-6, both are examples of NF-kB-upregulated targets directly involved in OM progression since they are responsible for amplifying the mucosal inflammatory response.^8 , 9^ Their concentration in saliva increases within 4 to 12 days after chemotherapy in adults; these levels were associated with the occurrence of oral lesions.^10^ Moreover, elevated levels of plasma TNF-α at the time of diagnosis have been associated with higher risks of developing OM during chemotherapy in acute lymphoblastic leukemia (ALL) pediatric patients.^11^

OM prevalence, severity, and form of manifestation varies in the pediatric cancer population,^12 , 13^ making it an interesting target for genetic analysis. Single-nucleotide polymorphisms (SNPs) in genes involving drug metabolism ( *ABCC2* , rs717620; *MTHFR* , rs1801133; *ABCG2* rs2231137) and cell signaling pathways ( *miR-1206* , rs2114358) have been associated with the incidence of chemo-induced OM in pediatric blood cancer patients.^14 – 17^ However, there are no reports on the relation between SNPs in genes involving antioxidant enzymes and chemo-induced OM in any population. Regarding polymorphisms in cytokine genes in the same context, reports are limited to studies involving adults.^18^

Rs4880 ( *SOD2* 47 C/T) is a commonly studied SNP that has been associated with diminished levels of SOD2^19^ ; which has been previously studied in the context of periodontal disease, being associated with susceptibility to treatment response.^20^ On the other hand, polymorphisms in the promoter region of the catalase gene ( *CAT* ) are rarely investigated in the context of oral diseases. Rs7943316 ( *CAT* −21 A/T), for example, is associated with *CAT* reduced expression^21^ and reports on the literature focus mainly on conditions such as obesity and vitiligo.^21 , 22^ For pro-inflammatory cytokines TNF-α and IL-6, SNPs associated with upregulation of gene expression, as well as elevated levels of circulating cytokine, are interesting targets in the context of OM. That is the case of rs1800795 ( *IL-6* −174 G/C)^23^ and rs1800629 ( *TNF-* α-308 G/A),^24^ both previously associated with oral diseases such as recurrent aphthous stomatitis^25^ and aggressive periodontal disease,^26^ respectively.

All in all, we have hypothesized that the aforementioned polymorphisms contributed to the establishment and aggravation of chemo-induced OM. This study sought to investigate the association of rs4880 ( *SOD2* 47 C/T), rs7943316 ( *CAT* −21 A/T), rs1800629 ( *TNF-* α −308 G/A), and rs1800795 ( *IL-6* −174 G/C) polymorphisms with the occurrence and severity of OM in children and adolescents with hematological cancers.

## Methodology

### Research ethics and study design

This study has been approved by the Research Ethics Committee of the Universidade Federal da Paraíba (UFPB) (CAAE: 64249317.3.0000.5188). All procedures were also in accordance with the 1964 Declaration of Helsinki and its later amendments or comparable ethical standards. A single-center, observational cross-sectional study was conducted with field sample collection and laboratory analysis. Sample and data collection from an accessible sample of unrelated subjects took place at the Hospital Napoleão Laureano (HNL), a reference facility for cancer treatment in João Pessoa, PB – Brazil, from July 2018 to March 2021. There was a standstill in sample collections during the period from March to December 2020, due to the COVID-19 pandemic.

Sample size was calculated according to Cohen (1988),^27^ considering an effect size H of 0.5 (mean), a type I error of 5%, a type II error of 20%, and a statistical power of 80%. The ideal sample size was estimated in 72 patients per group (with and without oral mucositis), considering a rate of patient loss of 15%. The study power analysis was conducted considering the frequencies of rs4880, rs7943316, rs1800629, and rs1800795 using the Online Sample Size Estimator (OSSE, Bioinformatics Institute of India, available at http://osse.bii.a-star.edu.sg/index.php) .

### Eligibility criteria and OM diagnosis

Children and adolescents included in this study were those with primary diagnosis of leukemia or lymphoma who were being, or had been previously, submitted exclusively to chemotherapeutical treatment and who had been assessed for oral alterations by the multidisciplinary oral care team during treatment. Exclusion criteria comprised patients without registries of oral examination by a calibrated professional of the oral care team during treatment, patients that were in no condition to perform sample collection (e.g.: in isolation, intubated, or severely debilitated) or whose caregiver did not consent to it, and those who were treated with a combination of chemo and radiotherapy.

All demographic information and data concerning the beginning of treatment and the occurrence and severity of OM were collected from the hospital's registration and management software *MV 2000* ( *MV Sistemas, Recife, PE – Brazil* ), as well as from appropriately-filled dental charts. In regards to OM diagnosis, the HNL multidisciplinary team chose the modified oral assessment guide (OAG) for detecting this condition, since it is an easily-applicable tool made for evaluating oral alterations related to antineoplastic treatments in children.^28^ It is composed of 8 points of assessment that are commonly affected by inflammation during cancer treatment (voice, swallowing, lips, tongue, saliva, labial/palate mucosa, labial mucosa, and gingiva) and for each point, a scale from 1 to 3 is used for grading the severity of inflammation: grade 1 indicates normality of appearance/function, grade 2 indicates mild or moderate OM, and grade 3 indicates severe OM.

To be considered for DNA sample collection, patients could be in any phase of their chemotherapeutical treatment (induction, remission, or maintenance). However, all data on OM diagnosis considered for our analysis referred to the initial phases of treatment (induction), which is when they are being monitored by HNL's oral care team and are usually submitted to high-dose chemotherapy, with higher chances of developing the condition.

For the OM occurrence analysis, patients were divided into two groups:

– **Group I** included individuals who did not present OM in the induction phase of leukemia or lymphoma treatment;
– **Group II** included individuals who developed OM in the same period, irrespective of its severity.
  For the OM severity analysis, only patients from group II with a thoroughly-filled OAG severity grading scale recorded in their dental charts were included. Our analysis considered the oral examinations performed in the first 60 days of treatment for each patient in an attempt to reduce bias due to treatment duration. Therefore:
– **Group III** included individuals with a mild case of OM or without OM in the first 60 days of treatment;
– - **Group IV** included individuals with SOM in the same period.

### Sample collection and DNA isolation

Oral mucosa cells were collected from a one-minute mouthwash with 6 mL of sterilized dextrose (3%) followed by an additional 3 mL of an ethanol-based buffer. For those who were too young and therefore unable to perform a mouthwash, oral epithelial cells were collected with aid of an Ayre spatula ( *Cralplast, Cotia-SP, Brazil* ).^29 , 30^

Each sample was centrifuged (3,000 rpm, 15 min) and the supernatant was discarded. The pelleted epithelial cells received 500 μL of lysis solution before storage at −20 °C. Genomic DNA was purified with ammonium acetate according to Aidar and Line^30^ (2007) and quantification was conducted in a Nanodrop 2000 spectrophotometer ( *ThermoFisher Scientific, Waltham-MA, USA* ). Samples were considered pure when the mean value of two OD 260/280 ratios were equal to 1.8 or higher.

### Genotyping of single-nucleotide polymorphisms (SNPs)

SNPs were selected using the dbSNP database ( http://www.ncbi.nlm.nih.gov/projects/SNP/ ) and were chosen based on functional significance. Minor allele frequency according to 1000Genomes database is 0.41 for rs4880, 0.48 for rs7943316, 0.09 for rs1800629, and 0.14 for rs1800795.

All SNPs were analyzed with the Polymerase Chain Reaction-Restriction Fragment Length Polymorphism technique (PCR-RFLP). Samples were amplified in 15 μL reactions containing 7.5 uL of GoTaq^®^ G2 Hot Start Green Master Mix ( *Promega Corporation, St. Madson - USA* ), 1 μL of each primer (10 μM), 1 μL of DNA, and nuclease-free water. Primer sets as well as the enzymatic digestion conditions were the same as previously described.^16 , 18 , 24^ Genotypes were analyzed through vertical electrophoresis in 6% polyacrylamide gels, followed by coloring with silver nitrate. The genotypes were identified by their band pattern according to literature.^21 , 25 , 31^ *SOD2* restriction fragments: CT (246, 157, 89 bp) and TT (157, 89 bp); *CAT* restriction fragments: TT (249 bp), AA (175, 74 bp), and AT (249, 175, 74 bp); *TNF-α* restriction fragments: GA (107, 87 bp), GG (107bp), and AA (87 bp); and *IL-6* restriction fragments: CC (117bp), GG (139bp), and GC (139, 117bp).

### Statistical analysis

For each polymorphism, the Hardy-Weinberg equilibrium (HWE) was obtained using the chi-squared (χ^2^) goodness-of-fit test. Demographic data were analyzed using Student's T test for continuous variables (age) and χ^2^ or Fisher's exact test for categorical ones (sex). For association analysis between genotype or allele frequency and OM occurrence/severity, χ^2^ and Fisher's exact tests were performed when appropriate. The odds ratio and corresponding confidence interval were calculated, when possible, for every association. Data analysis was conducted using the BioEstat 5.3 software ( *Instituto Mamirauá, Tefé-AM, Brazil* ) with a significance level of 5% and considering a confidence interval of 95%. Any p-values <0.05 were considered significant.

## Results

### Characteristics of the studied population

The sample was comprised of 95 patients ranging from 03 to 19 years of age (mean age = 10.36 ± 4.84 years), in which most were male individuals (57.89%). Six types of cancer were present in our sample and the most frequent type was ALL (74.73%) ( Table 1 ). After sample collection, the study power was estimated at 6.6% for rs4880, 14.8% for rs7943316, 8.3% for rs1800629, and 5.6% for rs1800795.

**Table 1:** Demographic and cancer type data of the studied population

Demography	Group I^1^	Group II^2^	P-value^1 , 2^	Group III^3^	P-value^1 , 3^	Group IV4	P-value^1 , 4^	P-value^3 , 4^
	(n= 15)	(n= 80)		(n=23)		(n=20)		
Age (years)	10,1 (±3.75)	10.4 (±5.04)	p>0.05 £	8,78 (±4.83)	p>0.05 £	10.95 (±4.75)	p>0.05 £	p>0.05 £
% boys	26,67	63,75	0.01 ≠ OR=4.83;	56,52	p>0.05 ≠	55	p>0.05 ≠	p>0.05 *
% girls	73,33	36,25	CI [1.41;16.57]	43,48		45		
Cancer type	ALL	AML	APL	CML	HL	NHL		
%	74.73	12.63	03.16	02.11	02.11	05.26		

ALL = Acute lymphoblastic leukemia; AML = Acute myeloid leukemia; APL = Acute promyelocytic leukemia; CML = Chronic myeloid leukemia; HL = Hodgkin's lymphoma; NHL = non-Hodgkin's lymphoma.

£ Student's T test;

≠ Fisher's Exact test;

* χ^2^ test.

Fifteen individuals did not present OM as an outcome during the induction phase of chemotherapy (group I), whereas 80 children and adolescents developed OM at some point during this period – representing 84.21% of the complete sample (group II). Out of this total, information concerning OM severity in the first 60 days of treatment was only available for 43 individuals. They were categorized into mild or no OM (group III, n=23) and severe OM (group IV, n=20).

When comparing groups I and II, significant differences were observed when considering the patients' sex: in group II, the observed frequencies for male and female were 63.75% and 36.25%, respectively; whereas in group I, they were 26.67% and 73.33% (p=0.01, Fisher's Exact test), respectively. Therefore, female patients were four times more likely to not present OM in the induction phase of chemotherapy when compared with male (OR=4.83; CI=[1.14;16.57]). No significant differences were observed when considering the patients' age for any of the groups (p>0.05, Student's T test). Table 1 shows the demographic data for the different groups.

### Polymorphism frequency and OM occurrence

Out of the 4 polymorphisms investigated in this research, only one, the rs4880 ( *SOD2* 47 C/T), was not in accordance with the HWE (p<0.05). Table 2 shows the genotypic and allele frequencies of all 4 polymorphisms for every association analysis.

**Table 2:** Genotypic and allelic frequencies for the OM occurrence analysis

	Group I^1^	Group II^2^	P-value^1 , 2^
	(n=15)	(n=80)	
	***SOD* 2 genotype** rs4880 (47 C/T)		
CC	0	0	0,3853 *
CT	09 (60%)	57 (71.25%)	
TT	06 (40%)	23 (28.75%)	
	Allele frequency (%)		
C	09 (30%)	57 (35.62%)	0.70033 ≠
T	21 (70%)	103 (64.38%)	
	***CAT* genotype** rs7943316 (-21 A/T)		
AA	01 (6.66%)	23 (28.75%)	0.1902 *
AT	10 (66.67%)	39 (48.75%)	
TT	04 (26.67%)	18 (22.5%)	
	Allele frequency (%)		
A	12 (40%)	85 (53.12%)	0.2624 *
T	18 (60%)	75 (46.88%)	
	***TNF-*** α **genotype** rs1800629 (-308 G/A)		
GG	11 (73.33%)	68 (85%)	0,4213 *
GA	04 (26.67%)	11 (13.75%)	
AA	0	01 (1.25%)	
	Allele frequency (%)		
G	26 (86.67%)	147 (91.87%)	0.3168 ≠
A	4 (13.33%)	13 (8.13%)	
	***IL-6* genotype** rs1800795 (-174 G/C)		
GG	10 (66.67%)	58 (72.5%)	0.8354 *
GC	04 (26.67%)	19 (23.75%)	
CC	01 (6.66%)	03 (3.75%)	
	Allele frequency (%)		
G	24 (80%)	135 (84.38%)	0.744506 ≠
C	6 (20%)	25 (15.62%)	

Group I = individuals who did not present OM during the induction phase of treatment. Group II = individuals who presented OM in the induction phase of treatment.

* χ^2^ test;

≠ Fisher's Exact Test.

**Figure 1:**
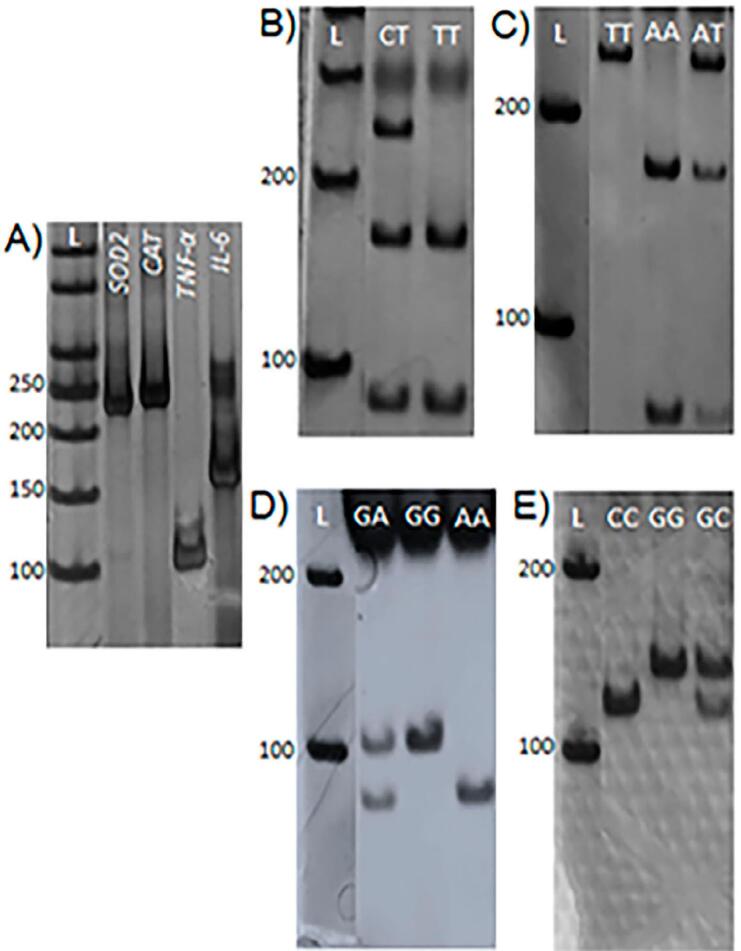
Representative fragments of PCR-RFLP reactions for each of the studied SNPs. A) Amplified fragments for each SNP: L – DNA ladder; *SOD* 2 (246 bp); *CAT* (249 bp); *TNF-* α (107 bp); *IL-6* (156 bp). B) *SOD* 2 restriction fragments: CT (246, 157, 89 bp) and TT (157, 89 bp); C) *CAT* restriction fragments: TT (249 bp), AA (175, 74 bp) and AT (249, 175, 74 bp). D) *TNF-* α restriction fragments: GA (107, 87 bp), GG (107bp) and AA (87 bp). E) *IL-6* restriction fragments: CC (117bp), GG (139bp), GC (139, 117bp)

Regarding the analysis between the polymorphism frequency and OM occurrence, groups I and II were considered. The comparison between groups did not show any significant differences (p>0.05). For *SOD2* , a higher frequency for the T allele and CT genotype was observed for both groups. For *CAT* , the AT genotype was the most frequent in the entire population and, although no significant differences were observed, the T allele was most frequent in the control group while the A allele prevailed in the OM group. For both *TNF-* α and *IL-6* , the G allele and the GG genotype were the most prevalent in the entire population. Figure 1 shows the band patterns after electrophoresis.

### Polymorphism frequency and OM severity

Regarding the analysis between the polymorphism frequency and OM severity, groups I, III, and IV were considered. Data on OM severity was available for 43 children, out of which 46.51% presented the severe form of the disease (n=20) ( Table 3 ).

**Table 3:** Genotypic and allelic frequencies for the OM severity analysis

	Group I^1^	Group III^2^	P-value^1 , 2^	Group IV^3^	P-value^1 , 3^	P-value^2 , 3^
	(n=15)	(n=23)		(n=20)		
		***SOD* 2 genotype** rs4880 (47 C/T)				
CC	0	0		0		
CT	09 (60%)	15 (65.22%)	1 ≠	16 (80%)	0.2661±	0.3271 ≠
TT	06 (40%)	8 (34.78%)		04 (20%)		
		Allele frequency (%)				
C	09 (30%)	15 (32.6%)	1 ≠	16 (40%)	0.4552 ≠	0.6263 ≠
T	21 (70%)	31 (67.4%)		24 (60%)		
		***CAT* genotype** rs7943316 (-21 A/T)				
AA	01 (6.66%)	6 (26.08%)		7 (35%)	0.04 * OR = 8.75 CI = [0.8;89]	
AT	10 (66.67%)	13 (56.52%)	0.3036 *	8 (40%)		0.5557 *
TT	04 (26.67%)	4 (17.40%)		5 (25%)		
		Allele frequency (%)				
A	12 (40%)	2 (54.35%)	0.3229 *	22 (55%)	0.3168 *	0.8756 *
T	18 (60%)	21 (45.65%)		18 (45%)		
		***TNF-*** α **genotype** rs1800629 (-308 G/A)				
GG	11 (73.33%)	18 (78.26%)		19 (95%)		
GA	04 (26.67%)	05 (21.74%)	1 ≠	0	0.03 *	0.0307 *
AA	0	0		01 (5%)		
		Allele frequency (%)				
G	26 (86.67%)	41 (89.13%)	0.9999≠	38 (95%)	0.3909 ≠	0.4418 ≠
A	4 (13.33%)	5 (10.87%)		2 (5%)		
		***IL-6* genotype** rs1800795 (-174 G/C)				
GG	10 (66.67%)	17 (73.91%)		18 (90%)		
GC	04 (26.67%)	6 (26.09%)	0.3318 *	02 (10%)	0.1915 *	0.2503 ≠
CC	01 (6.66%)	0		0		
		Allele frequency (%)				
G	24 (80%)	42 (86.95%)	0.1799 ≠	38 (95%)	0.0665 ≠	0.2754 ≠
C	6 (20%)	4 (13.05%)		2 (5%)		

Group I = individuals who did not present OM during the induction phase of treatment. Group III =individuals from group II with a mild case of OM or no OM in the first 60 days of treatment. Group IV = individuals from group II with severe OM in the first 60 days of treatment. For the OM severity analysis, only patients from group II with a thoroughly-filled OAG severity grading scale recorded in their dental charts were included.

* χ^2^ test;

≠ Fisher's Exact Test.

For *CAT* , the comparison between genotypic frequencies of groups I, III, and IV did not show significant differences. However, when partitioning the χ^2^ contingency table, we observed that the AA genotype was the one with higher effect on the outcome in group IV (individuals with SOM), when compared with individuals from group I (individuals without OM) (p=0.04, χ^2^ test; OR=8.75; CI=[0.8; 89]). Significant differences were observed for *TNF-* α. For the analysis involving groups I and IV, the GA genotype was more frequent among patients without OM (p=0.03, χ^2^ test). This genotype was also more frequent in group III when partitioning the χ^2^ contingency table of the group III x IV analysis (p=0.0307, χ^2^ test). For *SOD2* and *IL-6* , the most prevalent genotypes were CT and GG respectively (p>0.05) and no differences were detected. in Figure 1 shows the band patterns after electrophoresis.

## Discussion

OM is a side effect of cancer treatment, which has been associated with increased use of hospital resources and higher hospitalization costs.^2^ In our study, we evaluated four SNPs in genes related to oxidative stress management and inflammatory response, aiming to identify possible genetic biomarkers of OM occurrence and severity in children and adolescents with the most common types of childhood cancer: leukemias and lymphomas.^32^

Our study focused on exploring different genetic polymorphisms than those commonly analyzed in the context of chemo-induced OM, which are usually related to drug metabolism.^14 – 16^ To our knowledge, this is the first study investigating rs4880, rs7943316, rs1800629, and rs1800795 in a group of pediatric cancer patients with OM since it was not possible to obtain information about the distribution of these polymorphisms in other pediatric populations presenting this outcome. This demonstrates the importance of more studies in this area.

Regarding demographic data, our sample was considerably young, with a mean age of 10 years-old. OM was the observed outcome for approximately 85% of all individuals. Younger age is one of the factors associated with the onset of this condition and it has been stated that OM prevalence may reach frequencies of up to 90% when considering individuals under 12 years of age.^1 , 33^ The high prevalence of chemo-induced OM on children and adolescents also influenced the number of individuals included in group I (individuals without OM in the induction phase of treatment), which was lower than what had been estimated in our sample size calculation.

Other aspects of our sample included a higher prevalence of the female sex in group I and this variable was associated to a higher probability of not developing OM during the induction phase of treatment when compared to male individuals (OR=4.83; CI=[1.14; 16.57]). This was also observed in another study^16^ performed in the same cancer treatment facility, which suggests that this might be an intrinsic characteristic of the studied population. An epidemiological study by Feliciano, Santos and Pombo-de-Oliveira^34^ (2018) reports higher incidence of childhood cancer among male, while data from the Brazilian National Cancer Institute (INCA) reports the female sex as a factor related to better prognostics for childhood leukemia.^35^

In regard to the genes involved in oxidative stress management, it was observed that the rs4880 ( *SOD2* ) frequencies were not in accordance with the HWE, similarly to other studies involving Brazilian populations.^36 – 38^ The CT genotype and T allele for this SNP were the most frequent in our population. The allelic frequencies of group II are comparable to those of the hypercholesterolemia group (C=37.5%; T=62.5%) from a study by Duarte, et al.^39^ (2010) in which this SNP was associated to the condition and to lower MnSOD activity. Although no associations between this SNP and OM occurrence/severity were detected in our study, a reduced enzymatic levels in our population would be expected, leading to an increased susceptibility to oxidative damage, which might be associated with other SNPs in this gene or even with the epigenetic profile of *SOD2* .

The rs4880 consists of a C→T substitution at position 47 of the MnSOD gene and corresponds to a Ala16Val substitution in the signal peptide of the final protein, which alters its conformation and affects the enzyme's transportation from the cytoplasm to the mitochondrial matrix, being associated with diminished enzymatic activity.^19^ The enzymes superoxide and catalase represent the first line of defense towards oxidative damage inside living cells. MnSOD, the SOD isoform that uses manganese ions as enzymatic co-factors, acts as a catalyst for the dismutation reaction of superoxide ions O^–^ generated in the mitochondria into hydrogen peroxide (H_2_O_2_). The following step for complete ROS neutralization is carried out by catalase, which converts the H_2_O_2_ into water and molecular oxygen.^6^

For rs7943316 ( *CAT* ), we observed that the AA genotype was more frequent in the group with severe OM (p=0.04, χ^2^ test) when partitioning the contingency table, despite no significant differences being found for the allelic and genotypic frequencies between group I and II. The AA genotype for this polymorphism has been associated with cerebral stroke in hypertensive men^40^ and a study has recently shown that the dominant model AA *versus* AT + TT is associated with risk of preeclampsia.^41^ The rs7943316 consists of a A→T substitution in position 21 of *CAT* promoter region and is associated with diminished expression levels.^21^ It should be considered that the promoter region of *CAT* may be epigenetically modulated by CpG island hypermethylation when submitted to prolonged exposure to ROS.^42^ Therefore, higher levels of oxidative stress could still be observed even in the absence of the rare allele for rs7943316 due to epigenetic downregulation of *CAT* expression.

For the pro-inflammatory cytokines' genes, the GA genotype for rs1800629 ( *TNF* -α) was more frequent in patients without OM (group I; p=0.03, χ^2^ test) as well as in patients with a mild case of OM or no OM in the first 60 days of treatment (group III; p=0.0307, χ^2^ test) when compared with those with severe OM (group IV). The A allele was also slightly more frequent in group I (13.33%) when compared to group II (5%), but no significant differences were found. The observed p-values suggest that bearing the GA genotype is associated with not developing severe cases of OM. However, it was not possible to calculate the odds ratio or generate a confidence interval to support this association due to the null frequency of events (n=0) observed in the contingency table when assuming an over-dominant model (GA x GG + AA).

The rs1800629 consists of a G→A substitution at position 308 in the promoter region of *TNF* -α and is related to higher frequency of gene expression and higher levels of circulating cytokine, being previously associated with susceptibility to chronic periodontitis and diabetes mellitus type II.^24^ According to a review by El-Tahan, Ghoneim, and El-Mashad^43^ (2016) *TNF* -α has several reported SNPs in its promoter region that are mostly studied in the context of autoimmune diseases and act in either up or downregulation of gene expression. However, results regarding the role of these polymorphisms (including rs1800629) in this context are conflicting. All this considered, it is possible that the influence of an isolated SNP upon TNF-α gene expression could be surpassed by the combined effect of multiple single-nucleotide variations in its promoter region, which might interfere with the outcome of inflammatory diseases such as OM. Therefore, further studies assessing SNP interactions become an interesting perspective on the matter.

For rs1800795 ( *IL-6* ), a higher frequency of the non-rare G allele and GG genotype were observed for all groups. The relation between rs1800795 and oral diseases has been previously explored. A recent meta-analysis by Zhao and Li^44^ (2018) has suggested that the presence of the G allele for rs1800795 is a risk factor for periodontitis among Brazilians, increasing the risk of developing this condition by 34.9%. In this study, although not significant, the frequency of the G allele was higher in group IV when compared to group I (p=0.06). This data suggests that, perhaps with a larger sample, an association with the severity of the disease could be detected. The rs1800795 is a G→C substitution at position 174 of the *IL-6* promoter region, being associated with higher transcription frequency of the gene and increased levels of circulating cytokine.^23^ IL-6 is a pleiotropic cytokine known to participate in the transition from innate to acquired immune responses, playing an important role in monocyte recruitment and differentiation into macrophages, as well as B cell maturation.^45^

Since we have studied the side effect of a treatment of a rare condition (childhood cancer) in a single cancer treatment facility, a small sample size is expected, resulting in low study powers. Moreover, for patients treated with chemotherapy, length of treatment has been cited as an important factor when considering duration and severity of OM, among other aspects.^46^ Therefore, we limited our analysis to those patients who had thorough records of OM assessment with a complete OAG evaluation in the first 60 days of treatment in order to reduce bias due to drug accumulation. This resulted in small sample sizes that may not have been able to reflect possible associations. Other limitations of this study include relying on the correct filling of medical and dental charts by the multidisciplinary team as well as the lack of information concerning some of the patients. Another important point to discuss is that the Brazilian population is highly mixed with a unique proportion of Amerindian, European, and African ancestries and this can be a confounding factor. However, due to the nature of this population, isolating specific racial characteristics is a poor predictor of genomic ancestry in Brazilian individuals.^47 , 48^ In spite of this, the informative and exploratory aspect of the present study should not be disregarded since our data can guide further studies that aim to expand the genetic characterization of OM in children and adolescents. Therefore, more studies on OM severity with larger sample sizes are encouraged, particularly for rs7943316 (CAT −21) and rs1800629 ( *TNF-α* −308).

Genetic studies such as this are important contributions to precision medicine, a health care model which advocates for the construction of biological databases for each individual based on concepts of classical genetics, metabolomics, and aspects of the patient's clinical phenotype, enabling the establishment of more accurate lines of treatment and/or prophylaxis. This is a promising perspective that could be applied for reducing treatment toxicity and improving the patients' survival rate in the context of pediatric cancer.^49^

## Conclusion

Our results suggest a tendency to AA genotype for rs7943316 ( *CAT* −21 A/T) in the group with severe oral mucositis. Data on rs1800629 ( *TNF-* α −308 G/A), regarding disease severity, are inconclusive. No associations were detected for rs4880 ( *SOD2* 47 C/T) and rs1800795 ( *IL-6* −174 G/C) in pediatric cancer patients with chemo-induced oral mucositis.
